# Preferential attentional engagement drives attentional bias to snakes in Japanese macaques (*Macaca fuscata*) and humans (*Homo sapiens*)

**DOI:** 10.1038/s41598-018-36108-6

**Published:** 2018-12-11

**Authors:** Nobuo Masataka, Hiroki Koda, Takeshi Atsumi, Madoka Satoh, Ottmar V. Lipp

**Affiliations:** 1 Primate Research Institute, Kyoto University, Inuyama, Aichi Japan; 2 Developmental Disorders Section, Department of Rehabilitation for Brain Functions, Research Institute of National Rehabilitation Center for Persons with Disabilities, Tokorozawa, Saitama Japan; 3 Japan Society for Promotion of Science (JSPS), Chiyoda-ku, Tokyo Japan; 4 School of Psychology, Curtin University, Perth, Western Australia Australia

## Abstract

In humans, attentional biases have been shown to negative (dangerous animals, physical threat) and positive (high caloric food, alcohol) stimuli. However, it is not clear whether these attentional biases reflect on stimulus driven, bottom up, or goal driven, top down, attentional processes. Here we show that, like humans, Japanese macaques show an attentional bias to snakes in a dot probe task (Experiment 1). Moreover, this attentional bias reflects on bottom up driven, preferential engagement of attention by snake images (Experiment 2a), a finding that was replicated in a study that used the same methodology in humans (Experiment 2b). These results are consistent with the notion that attentional bias to snakes reflects on an evolutionarily old, stimulus driven threat detection mechanism which is found in both species.

## Introduction

The preferential detection of threats to survival is an important mechanism in the defensive armoury of any organism placed into a situation where the need for foraging competes with the risk of falling prey to a predator. However, threat detection mechanisms that are highly adaptive at one stage of evolutionary history may become vestigial and even maladaptive at a later one. In primates, the preferential attentional processing of snakes may provide an example for such a development. Snakes are a prime predator of non-human primates to the extent that the selection pressure exerted by snakes shaped the evolutionary development of the primate visual system^1^ as indicated by the presence of neurons in the medial and dorsolateral pulvinar which show larger and faster responses to images of snakes than to images of threatening conspecifics^2^. Although snakes pose a lesser threat to the survival of modern humans, fear of snakes remains highly prevalent, even in locations where risk of fatal encounters with snakes is minimal. A large scale Swedish survey, for instance, revealed that 5.5% of a mainly urban sample reported levels of fear of snakes that are clinically relevant and which the respondents described as unjustified, uncontrollable, and impairing their everyday activities^3^. This rate seems remarkable as there is only one venomous snake native to Sweden (*Vipera berus*) and fatalities after bites are extremely rare^4^.

The predisposition to detect and process images of snakes preferentially has been documented in humans and other primates in a number of different paradigms, most prominently in fear conditioning, visual search, and spatial attention tasks^5^. Fear conditioning is assessed by pairing pictures of snakes or control pictures with aversive outcomes. In humans, preferential fear conditioning to snakes as indexed by physiological responses is evident in that it resists extinction, is selective to aversive learning, can be acquired within a single trial of training, and resists cognitive interventions such as instructions that further unconditional stimuli will not be forthcoming (for reviews see^6,7^). Similar evidence for preferential learning of a selective association between snakes and negative outcomes has been shown in laboratory reared rhesus monkeys (*Macaca mulatta*), even in absence of a direct experience of the negative outcome (but^6,8^, see^9^ for a qualification).

Visual search has been employed as a paradigm to demonstrate preferential detection of snakes relative to non-threatening stimuli, such as flowers and mushrooms^10^ or non-threatening animals^11^. Here, participants are required to detect singleton pictures, e.g., a snake among flowers or a flower among snakes, as quickly as possible. Faster detection of snakes has been shown in human adults and children^12^ and in Japanese macaques (*Macaca fuscata*)^13,14^. Moreover, in human participants the faster detection of snakes is enhanced in participants who self-report high levels of fear of snakes^10,15^. This observation of individual differences renders it unlikely that preferential detection of snake pictures reflects on low level perceptual features of the stimulus materials used rather than on the content depicted. The evidence from visual search tasks has been criticised, however, as it is not clear whether faster detection of snake than of flower targets reflects on differences in target detection or on slower search through snake than through flower backgrounds^11^.

A second task that is used, in particular in clinical research, to assess preferential allocation of spatial attention or biased attentional processing is the dot probe task^16^. In this task, participants are simultaneously presented with two cues, e.g., pictures of a snake and a flower, for a short period of time and after their removal a probe, e.g., a dot, is presented in one of the cued locations. Participants are required to indicate the location or the identity of the probe as quickly as possible. Preferential allocation of attention to one of the images is indicated by faster responding to the probe that replaces this picture, e.g., attentional bias to snakes is evident if the probe replacing the snake is detected faster than the probe replacing the flower, whereas the inverse pattern of results is indicative of avoidance. The dot probe task has been applied to assess attentional bias not only in the context of animal phobias but anxiety disorders more broadly^17^ and more recently in contexts as diverse as addiction^18^, obesity and disordered eating^19^, and pain^20^. This broad interest reflects on the notion that attentional biases as reflected in the dot probe task may contribute to the development and maintenance of emotional disorders and that their reduction in attentional bias modification training may aid the treatment of these disorders^21^.

Past research using the dot probe task in human participants has provided evidence that pictures of snakes (or spiders) bias attention and are preferentially attended to and that this bias is larger in participants who reported higher levels of fear of snakes^22^. Whether a similar attentional bias to snakes in the dot probe task can be observed in non-human primates is currently unclear^23^. This knowledge gap seems surprising given the importance attributed to attentional biases for the maintenance and mitigation of emotional disorders. Thus, it is important to determine whether such biases can be found in non-human primates or whether they reflect on an evolutionarily relatively recent phenomenon and are limited to *Homo sapiens*.

While there is no work on attentional bias to snakes in non-human primates, there is some prior work that assesses attentional biases to social stimuli, faces of conspecifics, in macaques. Contrary to predictions, one study failed to find evidence for larger allocation of attention to faces of infants over faces of adults^24^. However, the remaining two studies reported attentional biases to threatening facial expressions using either faces with neutral expressions^25^ or scrambled faces^26^ as controls. Moreover, the former study confirmed that this bias was selective to threatening facial expressions and not evident when positive expressions were presented together with the neutral controls. The prior work on attentional biases in non-human primates confirms the utility of the dot probe paradigm for their assessment. Thus, Experiment 1 will employ the dot probe paradigm to confirm that, like humans, non-human primates (*Macaca fuscata*) display an attentional bias to snake images.

Although attentional biases in the dot probe task are a well-established phenomenon in humans, it is currently unclear which attentional processes underlie the biases observed^24^. Faster detection of a probe presented after a picture of a snake may reflect on preferential attentional engagement by snakes, i.e., the faster allocation of attention to snake images. Conversely, it may reflect on a delay of attentional disengagement from snake pictures after attention has been allocated to them. Whereas the former process would suggest a stimulus driven, bottom up attentional process the latter indicates the involvement of top down, goal directed attentional processes. Dissociating these two processes is important not only for a complete understanding of attentional biases, but also for the design of effective interventions as different interventions may differ in their effectiveness to modify them. It should be noted that some of the work on attentional biases in children^12^ can speak to this dissociation. However, past work in children has largely employed visual search tasks in which it is difficult to differentiate bottom-up and top-down attentional processes^11,15^.

Koster and colleagues^27^ suggested the inclusion of baseline trials on which two neutral images, e.g. two images depicting flowers, are presented as a means to dissociate preferential attentional engagement from delayed attentional disengagement in a dot probe task. In brief, preferential engagement is indicated if a probe in the same location as, say, a snake image (snake-valid cued) is found faster than a probe on a baseline trial whereas delayed disengagement is indicated if a probe presented in the location not occupied by the snake (snake-invalid cued) is found slower than a probe on a baseline trial.

Using pictures of mutilations and physical threat scenes, Koster and colleagues found that the attentional bias in their study was mainly driven by delayed disengagement of attention from threat, and a similar pattern emerged in a study that used words as stimulus materials^28^. However, more recent studies using a modified version of the dot probe task^29^ or a spatial cuing task^30^ also provide evidence for preferential attentional engagement by threatening visual stimuli. Experiment 2a will employ a design comprising snake-valid, snake-invalid, and baseline trials to assess whether the attentional bias in *Macaca fuscata* as shown in Experiment 1 is driven by preferential attentional engagement or delayed attentional disengagement. The study that documented attentional bias to snakes in humans^22^ did not employ an analysis that would permit the assessment of the underlying attentional processes, but reported performance on snake-valid and snake-invalid cued trials only (as well as spider-valid and spider-invalid cued trials). However, the study included baseline trials in which no animal pictures were presented. Thus, Experiment 2b will report a reanalysis of these data delineating whether the attentional bias to snake images is mediated by the same attentional processes in *Homo sapiens* and *Macaca fuscata*.

In summary, the current report is of two experiments conducted in non-human primates and a reanalysis of published data from humans. Experiment 1 was designed to determine whether Japanese macaques (*Macaca fuscata*) display an attentional bias to pictures of snakes relative to pictures of flowers in a manner similar to that observed in humans^22^. Experiment 2a included baseline trials on which two flower pictures were shown as cues and was conducted to determine whether attentional bias to snakes in Japanese macaques reflects on preferential attentional engagement to or delayed disengagement from snakes. Experiment 2b reanalysed published data to assess whether the attentional bias in *Macaca fuscata* and *Homo sapiens* reflects on the same underlying attentional process.

## Materials and Methods

### Subjects

#### Experiments 1 and 2a

Three female Japanese macaques (Neru, Anoi, and Ringo) aged 6, 7 and 8 years participated in the experiments. All were born in captivity in social groups at the Primate Research Institute of Kyoto University (Japan) and lived with their own mother and other group members in an enclosure that provides access to a large outside area and is located in a rural environment. Thus, prior encounters with snakes cannot be excluded completely, but can be assumed to be extremely unlikely. All experimental procedures were approved by the Ethics Committee of the Primate Research Institute of Kyoto University (#2015-012) and comply with the Guide for the Care and Use of Laboratory Primates (Third Edition, the Primate Research Institute, Kyoto University, 2010).

#### Experiment 2b

Sixty nine undergraduate students (13 men; age 17–40; *M* = 20.2, *SD* = 4.92) provided informed consent and volunteered in exchange for course credit. The study had been approved by the University of Queensland ethics review board (B/14/Psych/ARCL/99).

### Apparatus

#### Experiments 1 and 2a

The monkeys were individually tested and performed the experimental tasks in a custom-made experimental operant box (450 mm W × 450 mm D × 600 mm H) placed in a sound-attenuating chamber. A 21.5-inch touch-sensitive LCD screen (Dell, S2240T, USA, 1920 × 1080 pixels display resolution) was mounted on one side of the experimental box. A universal food dispenser (BUF-310-P100, BIOMEDICA, Osaka, Japan) was placed in the experimental box to provide a piece of sweet potato, apple or raisins as a food reward. The food dispenser was controlled by computers with USB I/O interfaces (2-Channel USB Powered Relay Module, Numato Lab, Numato Systems, Bangalore, India). Stimulus presentation and food dispensation were controlled by a custom-written computer program.

#### Experiment 2b

Participants were tested individually and performed the task on a personal computer running a custom written program. Stimuli were presented on a 17-inch colour monitor at a distance of 70 cm and participants provided responses via a button box.

### Stimuli

#### Experiments 1 and 2a

The current study employed nine pictures of snakes and flowers used in prior research with human participants^22^. All pictures were sized to an area of 195 × 260 pixels. The average luminance was adjusted to equivalent values.

#### Experiment 2b

In addition to the pictures of snakes and flowers, participants also saw nine pictures each of spiders and mushrooms^22^.

### Procedures

#### Experiment 1

**Figure 1:**
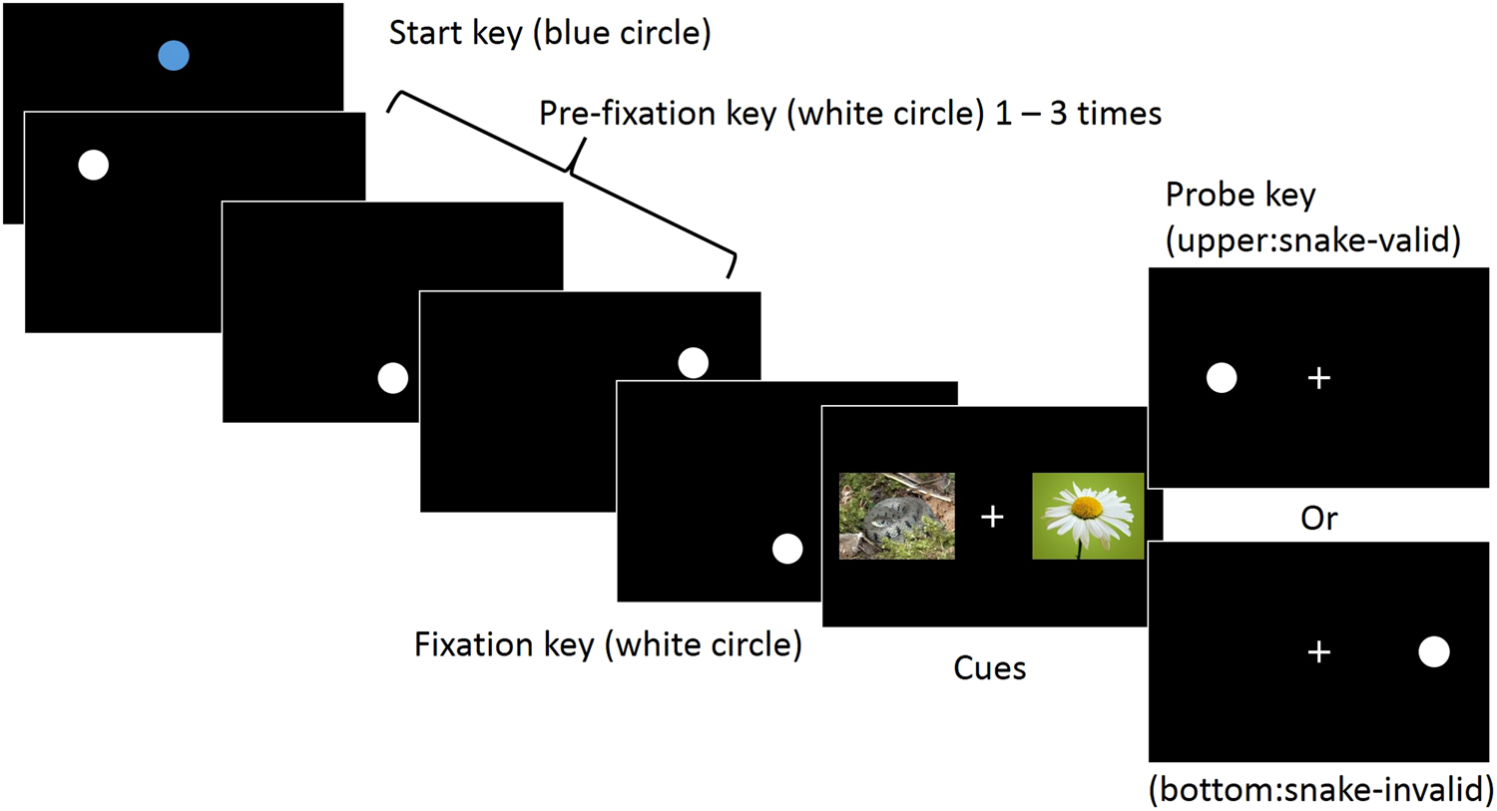
Event sequence for each trial of the dot-probe task used in Experiments 1 and 2a. The presentation of a blue start key was followed by one to three pre-fixation keys, the fixation key, two cue pictures and a probe key. Keys were presented until touched. Pictures were presented for 100, 300, or 500 ms.

Throughout the experiments, the monkeys were required to touch circles (size: 120-pixels in diameter) displayed one at a time on the touch screen. The events within a single trial and example pictures are illustrated in Fig. 1. At the start of each trial, a blue circle (start key) located at a position of 256-pixels above the screen center was presented. After the start key had been touched it disappeared and one to three white circles (pre-target keys) were displayed in random positions to be touched. Then, a white circle (fixation key) was displayed at a position of 154-pixels below the screen center. After touching, the fixation key disappeared and two pictures were presented in the positions of 410 pixels to the left or right of the screen center. In Experiment 1, each pair comprised one snake and one flower picture. The pictures were randomly selected from the nine snake and nine flower pictures. After a stimulus onset asynchrony (SOA) of 100, 300 or 500 ms a white circle appeared as the probe key in the location previously occupied by one of the pictures. The SOA was varied randomly from trial to trial within participants until 7 trials per SOA had been presented within each session. Multiple SOAs were selected to reflect the procedures used in past studies that employed the dot probe task in monkeys^24,26^. A fixation cross (size: 48 × 48 pixels) was shown in the screen center with the two pictures and the probe key following the procedure used in humans^22^. After the probe key was touched, the screen blacked out and the monkey was reinforced with a food reward accompanied by auditory feedback. The monkeys were required to touch the probe key within 1500 ms. After reinforcement, a 1000 ms inter-trial-interval (ITI) was inserted, and the next trial was started. If the monkey failed to touch the probe key within 1500 ms or touched the location opposite to the probe key, the screen blacked out and a buzzer sound was played. After an error, a 4000 ms ITI was inserted as negative feedback, and the next trial started delayed.

On snake-valid trials, the probe key appeared at the location previously occupied by the snake picture; on snake-invalid trials, the probe key appeared at the location previously occupied by the flower picture. Half of the trials in each session were snake-valid, whereas the other half were snake-invalid. Probe position (left or right) and SOA were counterbalanced across trials and each trial configuration was repeated 7 times. Consequently, each session comprised a total of 84 trials (2 × 2 × 3 × 7; Trial type [snake-valid, snake-invalid] × Position [left, right] × SOA [100, 300, 500 ms] × Repetition), presented in a randomized order. Each subject completed a total of 21 test sessions.

#### Experiment 2a

Experiment 2a replicated the general procedure of Experiment 1, but 30 baseline trials on which two flower pictures were presented were randomly inserted among 30 snake-valid and 30 snake-invalid trials. The inclusion of baseline trials permits the assessment as to whether a response time difference between snake-valid and snake-invalid trials reflects on preferential engagement of attention by snakes or delayed disengagement of attention from snakes^27^. Preferential attentional engagement by snakes is indicated by faster probe detection on snake-valid trials than on baseline trials. Delayed disengagement of attention from snakes is indicated by slower probe detection on snake-invalid than baseline trials. Each test session in Experiment 2a comprised 90 trials (3 × 2 × 3 × 5; Trial type [baseline, snake-valid, snake-invalid] × Position [left, right] × SOA [100, 300, 500 ms] × Repetition), and each subject completed 15 test sessions.

#### Experiment 2b

In brief, participants were presented with a random sequence of 160 trials, five blocks of 32 trials each comprising two repetitions of the 16 possible combinations of the four picture categories (snake, spider, flower, and mushroom; for further details see^22^). The probe followed the picture on the left for one exemplar of each combination and on right for the second. On each trial, a central fixation cross was presented alone for 500 ms, with two pictures centred in the left and right half of the screen for another 500 ms, and with a small white dot in the left or the right half of the screen for a further 500 ms. After the button corresponding to the probe location was pressed, an intertrial interval of 2000 ms commenced. Trials were presented in a random order without interruption. The task commenced with 10 practise trials and the instruction to work as quickly as possible while avoiding mistakes.

### Data analyses

#### Experiments 1 and 2a

Response times (RTs) were measured from the appearance of the probe key to the touch response. Trials on which the subject failed to touch the correct location, did not touch within 1500 ms or did not touch the screen precisely (e.g., double touches) were excluded from the analysis (6.9% in Experiment 1 and 9.7% in Experiment 2a). RTs within the experimental conditions were averaged for each session. To examine the presence of attentional biases, trial type (snake-valid or snake-invalid for Experiment 1; snake-valid, snake-invalid or baseline for Experiment 2a) × target position (left, right) × SOA (100, 300, 500 ms) mixed-effect model analyses of variance (ANOVAs) were conducted on RTs. Sessions were treated as random effects in the models. Post-hoc comparisons were conducted by calculating least square means and confidence intervals of differences between the conditions to be compared. The statistical analyses were performed separately for the three subjects, by using the lmerTest package with *F-*values based on the Satterthwaite’s approximation for denominator degrees of freedom (for the details about the statistical procedure, see https://cran.r-project.org/web/packages/lmerTest/index.html) developed for R version 3.2.3. Significance levels were set at *P* < 0.05. We additionally subjected the results of the present study to mini meta-analyses^31^, using the R package, Metafor (https://cran.r-project.org/web/packages/metafor/metafor.pdf)^32^ to confirm the reliability of the attentional bias across our experimental manipulations. We calculated summary effect sizes of comparisons between conditions from individual results by using a random-effects model (restricted maximum likelihood estimation).

#### Experiment 2b

RTs from trials with errors (pressing the wrong button) and outliers reflecting RTs that deviated by more than three standard deviations from a participant’s mean were excluded from the analysis. Error rates were low (3–5%) and further analysis yielded no significant results. RTs from snake-valid cued trials, snake-invalid cued trials, and baseline trials, defined as trials on which a flower and a mushroom image were presented, were averaged and subjected to a within subject ANOVA conducted in SPSS. Follow-up analyses were performed with paired *t*-tests. All datasets are available through the OSF (https://osf.io/fvc9q).

## Results

### Experiment 1

**Figure 2:**
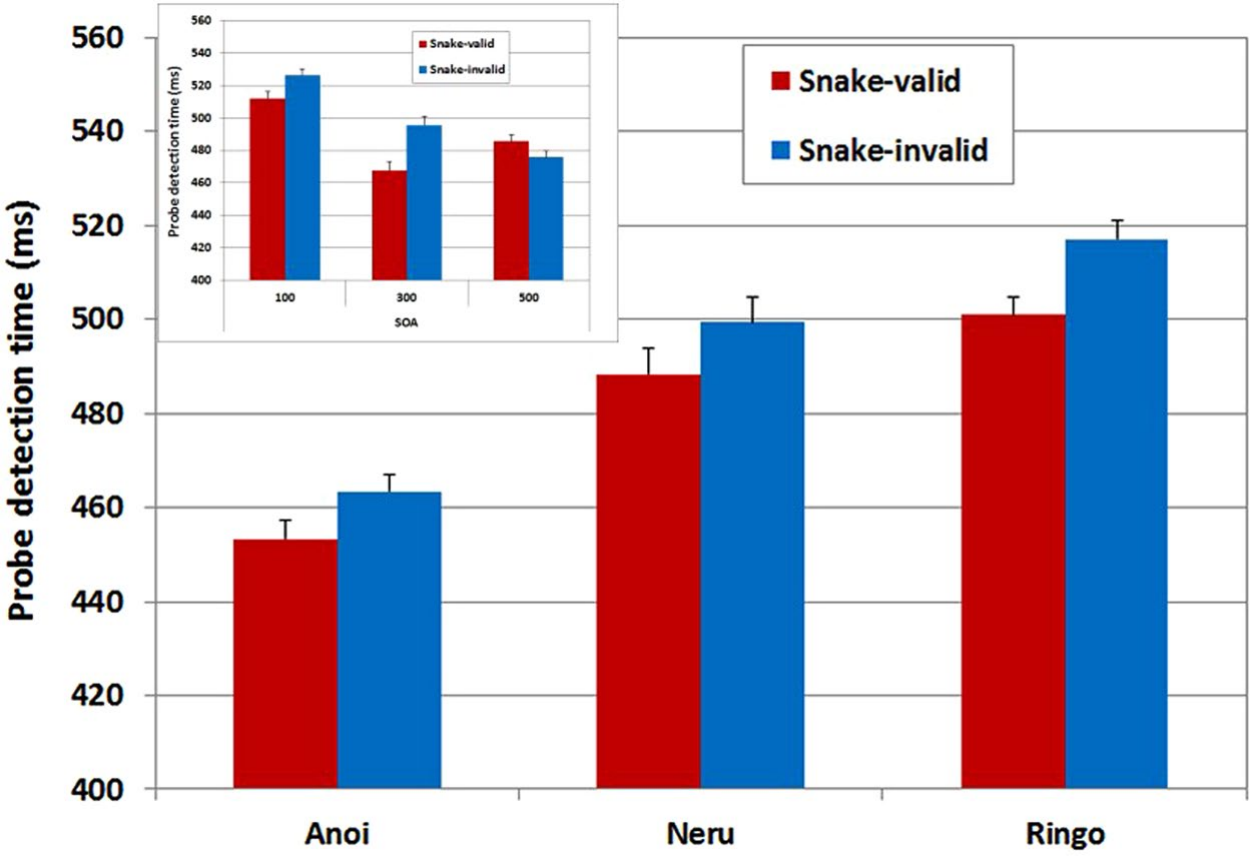
Probe detection time (ms) on snake-valid and snake-invalid trials of Experiment 1 for each subject averaged across stimulus onset asynchronies. The insert shows the performance of Neru as a function of SOA (error bars represent standard errors of the mean).

For all three subjects, response time decreased with increasing SOA (Neru: *F*(2,219.97) = 20.80, *p* < 0.001, ηp^2^ = 0.160; Anoi: *F*(2,219.97) = 27.10, *p* < 0.001, ηp^2^ = 0.200; Ringo: *F*(2,220) = 23.10, *p* < 0.001, ηp^2^ = 0.170) and all subjects were faster if the probe was presented on the side of their preferred hand, right for Anoi (*F*(1,219.97) = 27.80, *p* < 0.001, ηp^2^ = 0.110) and Neru (*F*(1,219.97) = 76.90, *p* < 0.001, ηp^2^ = 0.260), and left for Ringo (*F*(1,220) = 82.90, *p* < 0.001, ηp^2^ = 0.270). As can be seen in Fig. 2, probe detection was faster on snake-valid than on snake-invalid trials for Anoi (*F*(1,219.97) = 5.45, *p* *=* 0.020, ηp^2^ = 0.024) and Ringo (*F*(1,220) = 22.40, *p* < 0.001, ηp^2^ = 0.092), but not for Neru (*F*(1,219.97) = 3.83, *p* *=* 0.051, ηp^2^ = 0.017) for whom the analysis yielded a Trial type × SOA interaction (*F*(1,219.97) = 3.95, *p* *=* 0.021, ηp^2^ = 0.034). Follow up analyses confirmed that Neru was faster to detect the probe on snake-valid than on snake-invalid trials at a SOA of 300 ms (*t*(219.97) = 2.92, *p* = 0.004), but not at SOAs of 100 (*t*(219.97) = 1.49, *p* = 0.138) or 500 ms (*t*(219.97) = 1.01, *p* = 0.313). These results indicate that like humans Japanese macaques display an attentional bias to snakes. This bias was significant across all SOAs for two of the macaques tested whereas the third displayed the bias at a SOA of 300 ms only.

### Experiment 2a

**Figure 3:**
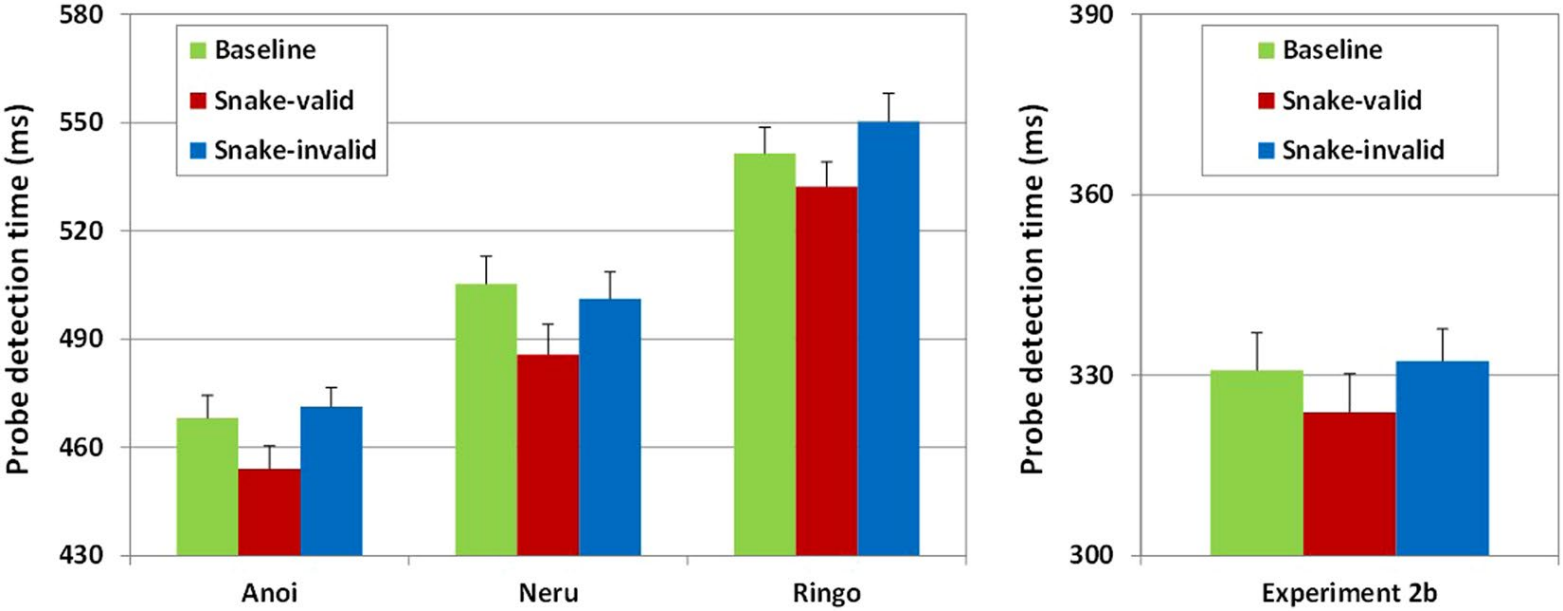
Probe detection time (ms) on baseline, snake-valid, and snake-invalid trials of Experiments 2a for each subject averaged across stimulus onset asynchronies (left panel) and of Experiment 2b (right panel; error bars represent standard errors of the mean).

As in Experiment 1, probe detection times decreased with increasing SOAs in all subjects (Neru: *F*(2,238.04) = 34.20, *p* < 0.001, ηp^2^ = 0.220; Anoi: *F*(2,238) = 51.20, *p* < 0.001, ηp^2^ = 0.300; Ringo: *F*(2,237.97) = 16.40, *p* < 0.001, ηp^2^ = 0.120), and all subjects were faster if the probe was presented on the side of their preferred hand, right for Anoi (*F*(1,238) = 79.80, *p* < 0.001, ηp^2^ = 0.250) and Neru (*F*(1,238.04) = 359.50, *p* < 0.001, ηp^2^ = 0.600), and left for Ringo (*F*(1,237.97) = 248.10, *p* < 0.001, ηp^2^ = 0.510). The probe detection times in the baseline, snake-valid, and snake-invalid trials of Experiment 2a are shown in the left panel of Fig. 3. Probe detection times were fastest on snake-valid trials yielding significant main effects for trial type for all subjects (Neru, *F*(2,238.04) = 3.09, *p* = 0.047, ηp^2^ = 0.025; Anoi, *F*(2,238) = 4.49, *p* = 0.012, ηp^2^ = 0.036; Ringo, *F*(2,237.97) = 5.56, *p* = 0.004, ηp^2^ = 0.045), but the overall pattern differed slightly. Subsequent post-hoc comparisons revealed faster probe detection on snake-valid than on snake-invalid or baseline trials for Neru (snake-invalid: *t*(238) = 2.25, *p* = 0.03; baseline: *t*(238) = 2.04, *p* = 0.04) and Anoi (snake-invalid: *t*(237.9) = 2.81, *p* = 0.005; baseline: *t*(237.9) = 2.30, *p* = 0.022). Ringo detected the probe faster on snake-valid than on snake-invalid trials, *t*(238) = 3.32, *p* = 0.001, with only a marginal difference between snake-valid and baseline trials, *t*(238) = 1.94, *p* = 0.054. There were no significant differences in probe detection time between snake-invalid and baseline trials for any of the three subjects (Neru: *t*(238) = −0.21, *p* = 0.830; Anoi: *t*(237.9) = −0.51, *p* = 0.610; Ringo: *t*(238) = −1.38, *p* = 0.170). This pattern of results seems to show that attentional bias to pictures of snakes in Japanese macaques reflects stimulus driven preferential engagement of attention.

### Experiment 2b

As shown in the right panel of Fig. 3, probe detection times differed across snake-valid, snake-invalid, and baseline trials, main effect for trial type, *F*(2,67) = 5.42, *p* = 0.007, ηp^2^ = 0.139, with faster probe detection on snake-valid than on baseline, *t*(68) = 2.79, *p* = 0.007, and on snake-invalid trials, *t*(68) = 3.15, *p* = 0.002, but no difference between baseline and snake-invalid trials, *t*(68) < 1. Thus, the attentional bias to snake pictures in humans, like in Japanese macaques, reflects on preferential engagement of attention.

### Mini meta-analyses

**Figure 4:**
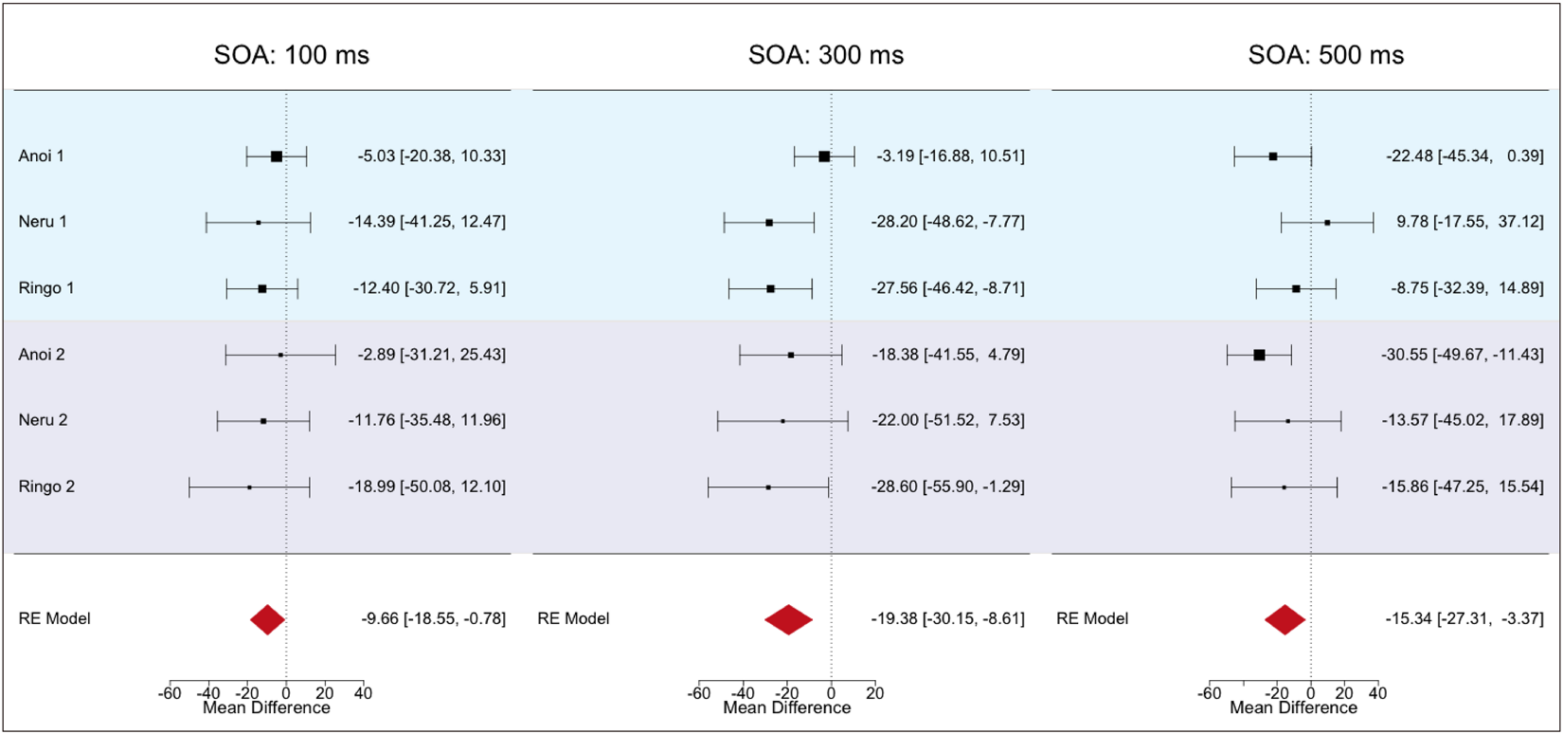
Forest plots showing individual and summary effect sizes from random effects meta-analyses for the difference in probe detection time (ms) between snake-valid and snake-invalid trials in Experiment 1 and 2a for each subject and SOA. Results of Experiment 1 are in the upper band and those of Experiment 2a are in the lower band. Black squares indicate mean time differences between trial types with corresponding 95% confidence intervals. Red polygons show the results from a random-effects model when integrating results across the two experiments.

The results of the current series of experiments seem to reflect a common trend of an attentional bias to snake images between species. However, the results of Experiments 1 and 2a for monkeys were sometimes weak and inconsistent. Unlike the other two monkeys, Neru showed a reduced RT on snake-valid trials only in one SOA condition in Experiment 1. On the other hand, comparisons between trial types in Experiment 2a revealed unique results for Ringo. To confirm the reliability of the attentional bias towards snake images in monkeys, we performed a mini meta-analysis across individual results. We firstly reassessed the bias towards snake images at each SOA by integrating individual results from Experiment 1 and 2a (N = 6). We calculated summary effect sizes for the comparison between snake-valid and snake-invalid trials at each SOA. Figure 4 shows the individual result estimates (RTs of snake-valid − snake-invalid condition) and the summary effect estimates (red polygons) at each SOA. Significant departures from a null effect (mean differences: MD = 0) were seen at all SOAs (100 ms: MD = −9.66, 95% confidence interval: CI = [−18.55, −0.78], *p* = 0.033; 300 ms: MD = −19.38, CI = [−30.15, −8.61], *p* < 0.001; 500 ms: MD = −15.34, CI = [−27.31, −3.37], *p* = 0.012). These results support the conclusion of faster RTs on snake-valid trials across SOAs in all monkeys.

**Figure 5:**
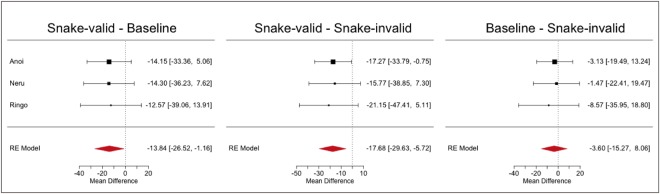
Forest plots showing effect sizes and summary effects from random effects meta-analyses for the difference in probe detection time (ms) for each comparison of trial types from Experiment 2a for each subject. The black squares show mean time difference between the trial types with corresponding 95% confidence intervals. Red polygons show the results from a random-effects model when integrating results across all 3 subjects.

Next, we conducted a second meta-analysis to assess the reliability of the attentional bias across the three trial types of Experiment 2a. We tested all combinations of trial types and found faster RTs in snake-valid than in the baseline and snake-invalid conditions (MD = −13.84, CI = [−26.52, −1.16], *p* = 0.033; MD = −17.68, CI = [−29.63, −5.72], *p* = 0.004, respectively) while no difference emerged between baseline and snake-invalid conditions (MD = −3.60, CI = [−15.27, 8.06], *p* = 0.55, Fig. 5). These results confirm the common trend of faster RTs in the snake-valid condition among the monkeys in the individual analyses of Experiment 2a.

## Discussion

Across two experiments which comprised three independent replications each, the current manuscript documents an attentional bias to snake images in Japanese macaques (*Macaca fuscata*). Moreover, Experiment 2a indicates that this bias reflects on preferential engagement of attention by snakes. In Experiment 1, all subjects were faster to detect probes following a snake picture than a picture depicting flowers, although for one subject this difference was significant only at one SOA. Experiment 2a, replicated this result for all subject across all SOAs. Moreover, the inclusion of baseline trials^27^ in Experiment 2a permits the delineation of the attentional process that underlies this bias. Rather than delayed disengagement of attention, the faster detection of probes following snake pictures reflects on the preferential engagement of attention by snakes. A reanalysis of existing data in humans^22^ based on a subset of trials that matched the trial types used in Experiment 2a confirmed that the same process underlies attentional bias for snakes in humans. Humans, like macaques, were faster to detect probes on snake-valid cued trials than on baseline trials or snake-invalid cued trials with no difference between snake-invalid cued trials and baseline trials. Rather than reflecting on delayed attentional disengagement, the attentional bias seen in humans is driven by preferential engagement of attention by snakes.

The current report indicates that, like humans, Japanese macaques show an attentional bias to images of snakes in the dot probe task. Moreover, it confirms across species that this attentional bias to snakes reflects on the preferential engagement of attention by snakes. This finding is consistent with the view that preferential attention to potential predators such as snakes is stimulus driven in a bottom up manner and may reflect on a perceptual system shaped by evolution that is highly sensitive to their detection^1,2^. It is in contrast with past findings from human research^27,28^ which suggest that attentional bias to stimuli depicting physical threat, pictures of mutilations or physical attack, or to negative words is driven by delayed disengagement of attention. Taken together the current results contribute to the increasing body of evidence that attentional biases may emerge on different levels of information processing which may require different approaches if they are to be the target of clinical interventions. This is well illustrated by the apparent failure of attentional bias modification training in cases of spider fear^21^.

The current results also raise questions as to the boundary conditions for the bottom up driven attentional bias observed across species in the current experiments. Past research in humans on attentional bias to threatening animals has frequently employed pictures of snakes and spiders as stimulus materials. Although not universally so^33^, most studies found similar results for both target animals with faster detection in visual search that is enhanced in persons fearful of either snakes or spiders^10,15^ or faster detection of probes cued by snakes or spiders^22^. However, a recent visual search study in Japanese macaques found faster detection of snakes but not of spiders^14^ and a human study employing a visual cuing paradigm found evidence for preferential bottom up attentional processing of spiders only if they were goal relevant^30^. This pattern of results suggests that more research is needed across experimental paradigms and participant species to gain a more complete understanding of the processes reflected in attentional biases to threatening animals.

## Conclusion

The current study provides converging evidence across two primate species for attentional biases to snakes in a spatial attention task. Moreover, in both species, the bias reflects on stimulus driven preferential attention to snakes rather than on delayed disengagement from snakes. This observation is consistent with the notion that attentional biases to snakes reflect on threat detection mechanisms that are evolutionarily old^1,2^ and found not only in non-human primates that share our evolutionary ancestors^5,6^. It enhances our understanding as to the processes that mediate attentional biases and suggests that attentional bias training to reduce fear of snakes has to target bottom up, stimulus driven attentional processes.
